# Vitamin C as an Adjuvant Analgesic Therapy in Postoperative Pain Management

**DOI:** 10.3390/jcm14113994

**Published:** 2025-06-05

**Authors:** Wioletta Mędrzycka-Dąbrowska, Sandra Lange, Sebastian Dąbrowski, Klaudia Długoborska, Renata Piotrkowska

**Affiliations:** 1 Department of Anaesthesiology Nursing & Intensive Care, Faculty of Health Sciences, Medical University of Gdansk, Dębinki 7, 80-211 Gdańsk, Poland; 2 Department of Internal and Pediatric Nursing, Medical University of Gdansk, Dębinki 7, 80-211 Gdańsk, Poland; langa94@gumed.edu.pl; 3 Department of Medical Rescue, Faculty of Health Sciences, Medical University of Gdansk, Dębinki 7, 80-211 Gdańsk, Poland; sebastian.dabrowski@gumed.edu.pl; 4 Department of Anesthesiology and Intensive Care Nursing, Medical University of Lublin, 20-059 Lublin, Poland; dlugoborska.klaudia@gmail.com; 5 Department of Surgical Nursing, Faculty of Health Sciences, Medical University of Gdansk, 80-211 Gdańsk, Poland; renata.piotrkowska@gumed.edu.pl

**Keywords:** ascorbic acid, antioxidant, pain, postoperative, analgesics

## Abstract

**Background/Objectives:** Postoperative pain occurs in approximately 80% of patients undergoing surgery. Although opioids remain the mainstay of postoperative pain management, their side effects have led to the development of multimodal analgesia strategies that aim to limit their use. Some studies have shown a correlation between vitamin C supplementation and a reduction in postoperative pain. The aim of this review was to describe the effect of vitamin C administration on postoperative pain intensity and opioid consumption. **Methods:** A systematic review was conducted in the fourth quarter of 2024. **Results:** Two authors systematically searched PubMed, CINAHL, Web of Science, and Cochrane Library databases. A total of 14 studies were included in the analysis. In these studies, the visual analog scale (VAS) was most often used to assess the postoperative pain intensity. In all studies, regardless of the measurement time, a reduction in the pain intensity was demonstrated compared to control or placebo groups. The analysis showed that intraoperative or preoperative vitamin C infusion reduced opioid consumption. The administered vitamin C doses ranged from 1 g to 3 g or 50 mg/kg intravenously during the perioperative period. **Conclusions:** The results showed a reduction in opioid requirements and pain intensity in patients receiving perioperative vitamin C, suggesting that vitamin C can be incorporated into multimodal postoperative analgesia strategies for surgical patients.

## 1. Introduction

Postoperative pain occurs in up to 80% of patients undergoing surgery [1]. Inadequate pain control after surgery can result in issues like pneumonia, acute coronary syndromes, sleep disturbances, and mood disorders, ultimately raising the risk of illness and death and lowering both well-being and patient contentment [2].

This may result in prolonged hospital stays and slower rehabilitation processes, and may also contribute to the development of chronic postoperative pain [1,2,3]. Despite extensive research efforts and the availability of effective analgesic measures, a significant therapeutic gap remains in treating the root causes of acute pain [4]. Various pain management strategies have been attempted with varying degrees of success. Until now, the focus has been on the development of opioid and non-opioid analgesics. Although opioids remain widely used, growing evidence highlights their potential for misuse, limitations, and adverse effects, including the risk of dependence and opioid-induced hyperalgesia (OIH) [3]. Currently, for the majority of patients, the most evidence-based approach appears to be a balanced multimodal analgesic strategy, using opioids at the lowest effective dose and for the shortest duration necessary. The aim of multimodal analgesia is to target pain through multiple pathways, optimize the balance between efficacy and side effects, and facilitate early opioid tapering [4]. Increasing evidence suggests that vitamin C may exhibit analgesic properties under certain clinical conditions, potentially alleviating suffering and improving patient quality of life [2,4,5,6]. Humans are unable to produce it internally, so it must be acquired from food sources. It serves as a cofactor for many mammalian enzymes and is differentially accumulated across various tissues and body fluids. Plasma and tissue concentrations of vitamin C are influenced by dietary intake, bioavailability, renal excretion, and metabolic utilization. All of these factors can be influenced by disease and may also vary based on body composition, genetic background, and potentially other factors such as physical activity [7].

The aim of this review was to describe the effect of vitamin C supplementation on postoperative pain levels and postoperative opioid consumption.

## 2. Methods

### 2.1. Study Design

This systematic review was conducted in the fourth quarter of 2024. Due to the significant heterogeneity of the available data, conducting a meta-analysis was not feasible. Therefore, a narrative synthesis approach was employed. This method primarily uses words and text to summarize and explain the findings of the synthesis [8].

### 2.2. Review Questions

The review was guided by the following PICO-based questions:

- Does vitamin C administration in surgical patients reduce postoperative pain intensity?
- Does vitamin C administration in surgical patients reduce postoperative analgesic consumption?
- What doses of vitamin C are effective at reducing the need for analgesics?

### 2.3. Search Strategy

Two authors systematically searched the following databases: PubMed, CINAHL, Web of Science, and Cochrane Library. The keywords used included “pain”, “acute pain”, “postoperative pain”, “vitamin C”, “ascorbic acid”, “pain level”, “pain relief”, and “opioids dose”. The keywords were combined using “AND” and “OR” operators.

All the publications were screened by title and abstract to exclude irrelevant entries. Any discrepancies were resolved through discussion among the researchers, and a final consensus was reached regarding the articles to be included. The Joanna Briggs Institute’s checklists for various study designs were used for a quality assessment [9]. The final search was conducted in October 2024.

Relevant studies were identified based on strict inclusion and exclusion criteria following the PICO framework (Table 1). Reviews were considered eligible if all the following criteria were met.

### 2.4. Study Selection

Using the PICO approach, our analysis considered studies presenting data on adult individuals (>18 years) undergoing surgery who received vitamin C supplementation (intervention). We excluded studies whose participants were children (<18 years) or non-surgical patients. We also excluded publications written in languages other than English and articles for which the full text could not be accessed.

### 2.5. Data Extraction

Data extraction was performed independently by two reviewers.

The extracted information included the following:

- The first author’s name;
- The year of publication;
- The study design;
- The number of patients;
- The types of surgical procedures;
- The vitamin C dosage and route of administration;
- The timing of administration;
- The main study outcomes.

The extracted data were recorded using Microsoft Excel.

### 2.6. Data Synthesis

Since a meta-analysis was not possible, a narrative synthesis approach was used to report the study findings.

This systematic review was conducted according to the PRISMA-SCR reporting guidelines and the Synthesis Without Meta-Analysis (SWiM) framework [10].

### 2.7. Assessment of Quality of the Included Studies

The studies’ quality was evaluated with the Joanna Briggs Institute checklist, modified to suit different research formats [9]. The articles were evaluated and scored based on their study type. The Newcastle–Ottawa Scale was used for a further quality assessment. The studies included in the review scored between 5 and 8 points (Table 2). The characteristics and key results from the selected studies are presented in Table 3.

## 3. Results

**Figure 1:**
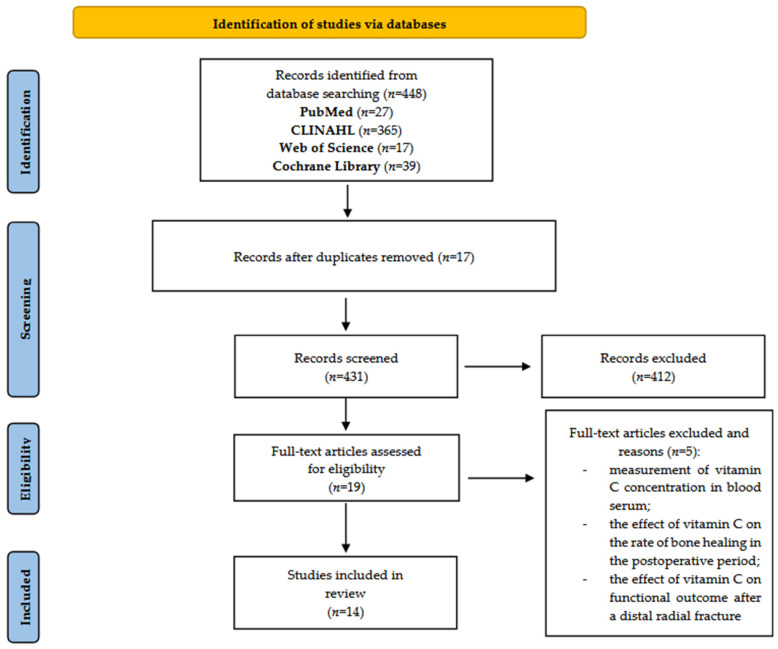
PRISMA flow diagram.

The initial database search identified 448 records. After eliminating duplicates and reviewing titles and abstracts, 29 articles were chosen for full-text evaluation. In the end, 14 studies fulfilled the inclusion criteria and were incorporated into the analysis. The study selection process is illustrated in the PRISMA flow diagram in Figure 1.

### 3.1. Vitamin C and Reduction in Postoperative Pain Intensity

The studies most commonly used the VAS scale to assess the pain intensity in the postoperative period [11,12,15,16,19,20,21,22,24]. Four studies employed the NRS [13,17,18,23]. Across all the studies, irrespective of when the postoperative pain intensity was measured, a reduction in pain intensity compared to the control or placebo groups was observed [11,12,13,14,15,16,17,18,19,20,21,22,23,24]. Pain was assessed at intervals of 30 min, 1 h, 6 h, 12 h, 24 h, and 48 h after surgery [11,12,13,15,16,17,18,19,20,21,22,23,24]. The study by Lee et al. used the Oswestry Disability Index (ODI) questionnaire and also observed reduced pain complaints over a more extended postoperative period [14].

### 3.2. Vitamin C and Reduced Analgesic Consumption Postoperatively

An analysis of the studies showed that vitamin C infusion during the preoperative or intraoperative period reduced the requirement for opioids (morphine, fentanyl) [12,13,15,17,18,20,21,22,23]. Three studies did not report postoperative opioid administration [14,16,19]. In the study by Sivro et al., metamizole was administered at a dose of 5 g, whereas in the study by Ayatollahi et al., paracetamol or pethidine was administered [11,24].

### 3.3. Vitamin C Formulation and Dosage

The vitamin C dosage in the reviewed articles ranged from 1 g to 3 g or 50 mg/kg intravenously during the intraoperative period, and was most frequently administered following anesthesia induction, lasting approximately 30 min [15,18,19,20,22,24]. The oral administration of vitamin C, both pre- and postoperatively, at a dosage of 500 mg twice daily also demonstrated a reduction in postoperative pain intensity [11,12,13,14]. The study by Moon et al. demonstrated a superior analgesic efficacy of vitamin C at a dose of 50 mg/kg combined with magnesium sulfate at 40 mg/kg in a single infusion compared to a vitamin C infusion alone [18]. A schematic representation of the above studies with regard to the analgesic effects of vitamin C can be found in Table 4.

## 4. Discussion

The effective management of postoperative pain significantly improves patient satisfaction, reduces postoperative complications, and shortens hospital stays [20]. The routinely employed methods to mitigate severe postoperative pain include nerve blocks and multimodal analgesia [16]. Pain intensity (PI) reduction is a measure of surgical treatment efficacy. The two most commonly utilized scales for assessing PI are the NRS and the VAS, which have been in use since the 1950s. Many studies have demonstrated significant similarities between these two scales, though direct interchange remains challenging. Despite numerous studies showing high correlations between the VAS and NRS, the NRS demonstrates a greater consistency and ease of use compared to the VAS. The widespread use of both scales sometimes leads to confusion between the VAS and NRS. This confusion disrupts the objective assessment and comparison of research findings. According to studies by Bielewicz et al., the NRS and VAS are not parallel scales, and they measure different aspects of pain [25,26]. The demand for ascorbic acid increases in postoperative patients because tissue injury leads to the substantial depletion of vitamin C reserves in the body [16]. The exact analgesic mechanism of vitamin C is not fully understood, although its antioxidant and neuromodulatory properties, which may play a role in pain relief, are often highlighted [27]. Many studies indicate that vitamin C supplementation in doses ranging from 0.5 to 3 g/day is effective at alleviating pain across various clinical conditions [4,11,12,13,14,15,18,19,20,22,24].

The suggested pathways through which vitamin C may help reduce postoperative pain include the following:

▪ Antioxidant activity: Vitamin C, a potent antioxidant, neutralizes free radicals that cause oxidative stress and tissue damage. Postoperative pain frequently involves inflammation and oxidative stress at the surgical site. By reducing oxidative stress, vitamin C may mitigate pain through minimizing tissue damage and inflammation [4].
▪ Inflammation modulation: Inflammatory processes significantly contribute to the development and continuation of pain following surgery. Vitamin C helps lower pro-inflammatory markers like IL-6, which trigger the release of acute-phase proteins. By influencing the body’s inflammatory response, vitamin C may ease discomfort and support tissue recovery [7].
▪ Support for connective tissue and healing: As a vital factor in collagen production, vitamin C strengthens tissue structure. Enhanced collagen formation can relieve nerve compression, thereby reducing discomfort. Faster and more effective tissue regeneration may also aid in pain control and speed up healing [4].
▪ Nerve protection: Vitamin C plays a role in preserving neural function. Nerve-related pain, including discomfort due to compression or irritation, often occurs after surgery. Thanks to its antioxidant and anti-inflammatory roles, vitamin C may help shield nerves and limit pain transmission [7].
▪ Neurotransmitter regulation: Vitamin C contributes to the production of catecholamine-based neurotransmitters. It is a required cofactor for dopamine β-hydroxylase, which transforms dopamine into norepinephrine, and may also enhance the dopamine output by maintaining tetrahydrobiopterin levels—an essential element for tyrosine hydroxylase activity. A similar mechanism supports serotonin synthesis. Medications that increase serotonin and norepinephrine levels are known to relieve pain [5].
▪ Novel analgesic mechanism involving vitamin C as a cofactor in the biosynthesis of amidated opioid peptides: Vitamin C serves as a cofactor for peptidylglycine α-amidating monooxygenase (PAM), which converts the terminal ends of peptide chains into their active amidated forms. Several of these amidated neuropeptides act on opioid receptors. For instance, endomorphin 1 and 2 and amidated tetrapeptides display an exceptional selectivity and affinity for the μ-opioid receptor. Interestingly, tissues involved in neurotransmitter and peptide hormone synthesis have particularly high concentrations of vitamin C [6].

A meta-analysis conducted as early as 2008, comprising five randomized studies, demonstrated that perioperative oral supplementation with vitamin C (200–1500 mg/day) up to 50 days post-surgery was associated with a reduced risk of complex regional pain in arms or legs post-surgery [28]. Recent studies similarly indicate positive effects on pain reduction, lower analgesic requirements, and an improved quality of life in patients receiving high-dose vitamin C supplementation (4 g/day). Significant reductions in secondary pain have also been observed in individuals administered 5 g intravenously per week or oral doses between 500 and 4000 mg [4]. Suzen et al. found that low plasma vitamin C levels post-surgery were associated with increased postoperative analgesic consumption [29].

Moon et al. administered vitamin C intravenously at 500 mg twice daily, given its low oral absorption rate and short plasma half-life. The oral absorption rates are approximately 63% at 500 mg and 46% at 1250 mg doses. The steady-state plasma vitamin C concentrations rarely exceed 80 μmol/L due to rapid renal clearance. Additionally, gastrointestinal issues, such as osmotic diarrhea, have been reported with oral vitamin C doses above 5 g [18].

A significant limitation of many studies on vitamin C and pain is an inadequate study design, primarily due to a general misunderstanding of vitamin C pharmacokinetics. Oral vitamin C absorption occurs through sodium-dependent vitamin C transporters (SVCT-1), and its efficiency declines at higher doses because of transporter saturation. A 200 mg dose of vitamin C is fully absorbed, whereas the absorption decreases to <75% and <50% at 500 mg and 1250 mg, respectively. In contrast, intravenous administration, which bypasses intestinal absorption regulation, achieves plasma concentrations up to 250 times higher. However, vitamin C has a short plasma half-life (~2 h), suggesting that the ideal approach to maximize absorption and plasma concentrations would be administration in several smaller doses throughout the day [5].

A 2022 meta-analysis by Suter et al. evaluated the efficacy and safety of perioperative vitamin C administration in adult patients undergoing non-cardiac surgery. It concluded that postoperative pain and morphine consumption could be reduced with perioperative ascorbic acid supplementation, although the evidence certainty was very low [30]. Across the reviewed studies, IV administration (50 mg/kg or 1–3 g boluses) during or shortly before surgery seemed most effective for acute pain control, suggesting that timing vitamin C to coincide with the peak oxidative and inflammatory stress of surgery could be critical for efficacy.

Intravenous vitamin C is generally well tolerated. However, increasing doses carry a higher risk of adverse effects, such as oxalate nephropathy, hemolysis in individuals with a G6PD deficiency, pseudohyperglycemia, and hypernatremia—particularly with rapid or repeated infusions. The main contraindications to high-dose vitamin C administration appear to be severe renal failure, a G6PD deficiency, and nephrolithiasis. Interference with glucometers may lead to erroneous therapeutic decisions, especially in patients receiving insulin therapy [31]. Intravenous vitamin C does not interfere with the action of anesthetic drugs and, in many cases, has beneficial effects as an adjuvant [32]. The pharmacokinetic interactions are generally not clinically significant. Vitamin C does not alter the metabolism of drugs used during anesthesia, such as propofol, volatile anesthetics, fentanyl, or rocuronium. Certain exceptions include urine acidification, which may prolong NSAID elimination (of marginal importance), and the induction of acidosis with megadoses of unbuffered ascorbic acid, which—in theory—may potentiate a rocuronium-induced neuromuscular blockade. In contrast, the pharmacodynamic interactions are mostly positive: vitamin C reduces the need for anesthetics and analgesics due to its analgesic properties, allowing for lower doses of propofol [33]. The price of vitamin C preparations varies depending on the formulation (oral or intravenous form) and the country in which they are sold. On the U.S. market, the difference in the price of 1 g of vitamin C between oral supplements and the only FDA-approved intravenous preparation is approximately one hundredfold [34,35]. In Europe, the prices of vitamin C products are significantly lower. Recent years have seen vitamin C shortages, especially in low-income countries due to global supply disruptions and rising demand, e.g., during the COVID-19 pandemic. China produces over 90% of the world’s vitamin C, so production cuts (e.g., in 2017 due to environmental regulations) and pandemic-era factory slowdowns led to global supply constraints and price spikes. These price increases caused shortages in countries like India, where government-imposed price caps made vitamin C tablet production unprofitable. Overall, access to vitamin C in low-income countries remains fragile and highly susceptible to global market and logistical shocks [36,37].

## 5. Limitations

There are several limitations of this systematic review. The heterogeneity of the included studies, in terms of surgical procedures, patient populations, vitamin C doses (500 mg to 3 g or 50 mg/kg), routes (oral vs. IV), and timing (pre-, intra-, and postoperative), makes direct comparisons difficult. Most studies did not assess the patients’ preoperative plasma vitamin C levels, which could have influenced their response to supplementation and confounded the findings. Most studies did not comprehensively report adverse events, particularly in high-risk populations (e.g., renal impairment, G6PD deficiency). These limitations underscore the need for larger, high-quality trials to determine the optimal dosing, timing, and true efficacy of perioperative vitamin C in diverse surgical settings.

## 6. Conclusions

While several studies have demonstrated reductions in postoperative pain or morphine consumption, and antioxidant depletion during surgery is well documented, large-scale randomized trials often show small or inconsistent effects, with some meta-analyses noting an uncertain impact on major outcomes. Insufficient high-quality evidence seems to be the reason why major anesthesiology and perioperative care guidelines do not currently recommend routine vitamin C administration. Future research should focus on large, well-designed randomized controlled trials in surgical populations, particularly in cardiac and orthopedic surgery. There is a need to explore vitamin C supplementation in high-risk or special surgical populations like elderly patients or those with a baseline vitamin C deficiency (malnourished, smokers, diabetics). Future research should also compare head-to-head timing regimens in controlled trials to find the most effective dosing strategy.

## Figures and Tables

**Table 1:** PICOS framework, inclusion and exclusion criteria, and search strategies.

	Inclusion Criteria	Exclusion Criteria
Population (P)	Surgical patient Patient ≥ 18 years old	Not a-surgical patient Patient < 18 years old
Intervention (I)	Vitamin C	-
Comparison (C)	❖ Placebo ❖ Standard pharmacological interventions	-
Outcome (O)	Pain level/opioid dose	-
Study Type (S)	❖ Quantitative studies ❖ Intervention studies (RCTs, non-RCTs) ❖ Observational studies ❖ Case study reports	❖ Reviews ❖ Letters to the editor
Years Considered/Time Period	All evidence published in the last 10 years, period: 2014–2024	Publications prior to 2013
Language	English	Other languages
Databases	MEDLINE (PubMed), CINAHL, Cochrane Library	Other databases
Keywords	“pain”, “acute pain”, “postoperative pain”, “vitamin C”, “ascorbic acid”, “pain level”, “pain relief”, “opioids dose”	n/a
Search Strategy	MEDLINE (PubMed): (“pain” OR “acute pain” OR “postoperative pain”) AND (“vitamin C” OR “ascorbic acid”) AND (“pain level” OR “pain relief” OR “opioids dose”) Limit: Language Results: 25 CINAHL: TX (“pain” OR “acute pain” OR “postoperative pain”) AND TX (“vitamin C” OR “ascorbic acid”) AND TX (“pain level” OR “pain relief” OR “opioids dose”) Limit: Language, years Results: 365 Web of Science: (“pain” OR “acute pain” OR “postoperative pain”) AND (“vitamin C” OR “ascorbic acid”) AND (“pain level” OR “pain relief” OR “opioids dose”) Limit: Years Results: 14 Cochrane Library: TX (“pain” OR “acute pain” OR “postoperative pain”) AND TX (“vitamin C” OR “ascorbic acid”) AND TX (“pain level” OR “pain relief” OR “opioids dose”) Limit: Years Results: 44	n/a

n/a—not applicable.

**Table 2:** Quality assessment of the included studies by the Newcastle–Ottawa Scale (NOS).

First Author, Year	Study Design	Selection	Comparability	Outcome	Total Scores
Ayatollahi V., et al. (2016) [11]	Arandomized double-blind clinical trial	***	*	***	7
Kumar A.A., et al. (2016) [12]	A prospective randomized double-blind study	***	**	***	8
Jeon Y., et al. (2016) [13]	A randomized controlled trial	***	*	**	8
Lee G.W., et al. (2017) [14]	A prospectively randomized trial	***	**	**	7
Jarahzadeh M.H., et al. (2019) [15]	A double-blind randomized study	****	**	***	8
Jain S.K., et al. (2019) [16]	A prospective randomized trial	**	**	**	6
Moon S., et al. (2019) [17]	A double-blind randomizedcontrolled trial	**	**	***	7
Moon S., et al. (2020) [18]	A double-blindrandomized controlled trial	***	**	***	8
Trankle C.A., et al. (2020) [19]	A prospective randomized double-blind study	**	*	**	5
Tunay D.L., et al. (2020) [20]	Arandomized double-blind clinical trial	***	***	***	8
Aboelela M., et al. (2023) [21]	Arandomized double-blind clinical trial	**	**	***	7
Han G., et al. (2024) [22]	A prospective randomized double-blind trial	***	**	***	8
Bala R., et al. (2024) [23]	A prospective randomized double-blind trial	**	**	***	7
Sivro M., et al. (2024) [24]	A prospective, single-blind, controlled, randomized clinical trial	**	**	***	7

* Each study may receive up to one star per numbered criterion within the Selection and Outcome domains (with a maximum of 4 stars for the Selection, 2 for the Comparability, and 3 for the Exposure/Outcome categories).

**Table 3:** Characteristics of included studies and results.

Author, Year	Country	Study Design	Number of Patients (*n*)	Surgical Procedures	Dosage and Route	Time of Administration	Pain Level	Results	Opioid Dose/Non-Opioid
Ayatollahi V., et al. (2016) [11]	Iran	A randomized double-blind clinical trial	40	Uvulopalatopharyngoplasty (UPPP) with tonsillectomy	An amount of 3 g of vitamin C in 500 mL of Ringer or 6 mL of normal saline in 500 mL of Ringer.	During the first 30 min after the beginning of the surgery.	The VAS scores at 6, 12, and 24 h after surgery were lower in the group receiving vitamin C than in the placebo group.	This research found that giving 3 g of intravenous vitamin C during surgery lessened pain after the procedure without raising adverse effects in patients undergoing surgery (UPPP and tonsillectomy).	Only IV paracetamol or pethidine.
Kumar A.A., et al. (2016) [12]	The Indies	A prospective randomized double-blind study	200	Laparoscopic surgeries	Group 1 received oral vitamin C (2 g) and group 2 patients received a placebo.	In the night and 2 h before surgery.	The VAS score in patients from group 1 receiving vitamin C immediately after surgery was 2.64 ± 1.13, compared to 3.72 ± 1.63 in group 2. At the 30th minute, the VAS score was 2.14 ± 0.51 in group 1, while it was 2.88 ± 1.07 in group 2.	We found that vitamin C use lessened immediate postoperative pain and decreased the need for fentanyl as a rescue pain reliever after surgery.	An injection of fentanyl, 25 micrograms IV, was given as rescue analgesic when the patient had a VAS score > 4.
Jeon Y., et al. (2016) [13]	Korea	A randomized controlled trial	100	Laparoscopic colectomy	Vitamin C, 50 mg/kg (ascorbic acid, 10 g/20 mL), mixed with 0.9% NaCl to reach a total volume of 50 mL; the participants in the placebo group were given normal saline alone.	The solution was infused over 30 min using an infusion pump.	The NRS pain scores during coughing and the NRS fatigue scores were similar across both groups at each measured time point.	This study demonstrates that a high-dose vitamin C infusion lowered pain within the first 24 h after surgery and reduced early postoperative morphine use. Further investigation is required to determine if increasing the dose and extending the infusion duration can enhance these outcomes.	All patients began patient-controlled analgesia (PCA) upon reaching the post-anesthesia care unit (PACU). The PCA delivered 1 mg of morphine boluses with a 5 min lockout and no continuous infusion. Patients were advised to press the PCA button whenever they felt pain. If the pain persisted above a VAS score of 4 for at least 30 min, a 50 mg tramadol injection was administered as additional relief.
Lee G.W., et al. (2017) [14]	Korea	A prospectively randomized trial	123	Posterior lumbar interbody fusion (PLIF)	Vitamin C in pills or placebo pills (no dose information).	Vitamin C therapy began on the first day after surgery and was given each morning for the next 45 days.	The clinical outcome was assessed using the Oswestry Disability Index (ODI). The intensity of lower back pain improved significantly in both groups relative to the pre-surgery pain levels; however, no notable differences emerged between the groups throughout the follow-up phase.	The pain intensity one year after surgery, the main outcome, did not differ significantly between the two groups. However, vitamin C might support better functional recovery following PLIF surgery, particularly within the initial 3 months after the operation.	No data.
Jarahzadeh M.H., et al. (2019) [15]	Iran	A double-blind randomized study	70	Laparoscopic surgery	An amount of 2 g of vitamin C mixed with 0.9% NaCl (500 mL) or only 500 mL of 0.9% NaCl.	A total of 30 min after induction of anesthesia injection.	The mean VAS scores (±standard deviations) at 1 h were 2.09 ± 2.44 and 3.54 ± 2.44 (*p* = 0.011); at 6 h, they were 1.66 ± 1.84 and 3.34 ± 2.04 (*p* = 0.001); and at 24 h, they were 1.11 ± 0.57 and 1.61 ± 1.6 (*p* = 0.001) for the experimental and control groups, respectively.	The use of vitamin C reduced the incidence of a sore throat and the pain score. Vitamin C appears to reduce the need for morphine to manage postoperative pain.	A total of 5 mg of morphine after the induction of anesthesia.
Jain S.K., et al. (2019) [16]	The Indies	A prospective randomized trial	60	Patients with isolated foot and ankle trauma	Patients with isolated foot and ankle trauma who underwent surgery were randomly allocated to receive either 500 mg of vitamin C or a placebo tablet, taken twice daily.	All patients additionally received 75 mg of diclofenac sodium twice daily for five days to manage acute pain and reduce inflammation, and later as needed.	The group that received vitamin C showed improvement in the VAS scores at the end of the second and sixth weeks of follow up, reduced analgesia requirements, and an improved functional outcome compared to the placebo group.	This study shows that the supplementation of vitamin C in patients undergoing surgery for foot and ankle trauma helped to reduce the analgesic requirements, improve the VAS scores, and achieve better functional outcomes.	No data.
Moon S., et al. (2019) [17]	Korea	A double-blind randomized controlled trial	60	Elective laparoscopic hysterectomy	An amount of 500 mg of vitamin C mixed with 0.9% NaCl (500 mL) or only 500 mL of 0.9% NaCl.	Twice a day from 7 a.m. and 7 p.m. on the day of surgery to the third day after surgery.	The NRS score of PLSP showed a statistically significant difference (*p* < 0.001), particularly 24 h after the operation (*p* = 0.002).	The total fentanyl use after surgery was notably lower in group C at both 24 and 48 h post-operation (*p* = 0.002 and *p* = 0.012, respectively). Group C also reported a significantly reduced PLSP intensity at 24 h (*p* = 0.002). Moreover, the occurrence of PLSP was considerably less in group C at both time points (*p* = 0.002 and *p* = 0.035, respectively).	Following surgery, a PCA device was attached to the IV line, and patients were moved to the post-anesthesia care unit (PACU). The PCA solution included 10 μg/mL of fentanyl in saline, delivering 1 mL bolus doses with a 5 min lockout period and no continuous background infusion.
Moon S., et al. (2020) [18]	Korea	A double-blind randomized controlled trial	132	Laparoscopic gynecology operation	There were 4 groups: Group M: magnesium sulfate, 40 mg/kg; Group V: vitamin C, 50 mg/kg; Group MV: magnesium sulfate + vitamin C—magnesium sulfate, 40 mg/kg, and vitamin C, 50 mg/kg; Group C: control group —0.9% NaCl, 40 mL.	No data.	The pain scores at rest after surgery were significantly lower in group MV at 1, 6, 24, and 48 h (4\[3, 4]; 3\[2, 3]; 2\[1, 2]; and 1.5\[1, 2], respectively) compared to those of group C (5\[4, 6]; 3\[3, 5]; 3\[2, 5]; and 3\[1.75, 4], with *p*-values of 0.001, 0.001, <0.001, and 0.002). At the 1 h mark, group V also showed significantly lower scores (4\[3, 4]) than group C (5\[4, 6]; *p* = 0.006). Moreover, group MV had markedly lower NRS scores than both group M and group V at 24 h post-surgery (*p* < 0.001 and *p* = 0.002, respectively). No significant differences in pain scores were observed between group M and group V.	The combined use of magnesium sulfate and vitamin C offers added value in managing postoperative pain following laparoscopic gynecologic procedures when compared to the use of either magnesium sulfate or vitamin C alone.	The PCA mixture consisted of 15 μg/mL of fentanyl in saline, set to deliver 1 mL bolus doses with a 5 min lockout and no continuous infusion. Following surgery, the PCA device was attached to the IV line, and patients were moved to the post-anesthesia care unit (PACU). If additional pain relief was needed, 30 mg of ketorolac was given upon request.
Trankle C.A., et al. (2020) [19]	USA	A prospective randomized double-blind study	20	Catheter ablation (treatment for atrial fibrillation (AF)	Intravenous vitamin C (50 mg/kg administered) or matched placebo.	Administered every 6 h for a total of four doses, starting with one given prior to the ablation procedure.	No data (only level/changes in CRP, IL-6, and ascorbate).	High-dose ascorbic acid is safe and generally well tolerated during AF ablation and may help reduce the increase in C-reactive protein, though no consistent effects were observed on interleukin 6 levels. Additional research is required to confirm these results and assess its potential to improve meaningful clinical outcomes.	No data.
Tunay D.L., et al. (2020) [20]	Turkey	A randomized double-blind clinical trial	165	Elective major abdominal surgery	Group M (*n* = 55) was given 6 mg of melatonin (Melatonina 3 mg tablets), group C (*n* = 55) received 2 g of vitamin C (Solgar Vitamin C 1000 mg tablets), and group P (*n* = 55) was administered a placebo tablet, all taken orally in the preoperative area.	One hour before the surgery.	The postoperative VAS scores were notably reduced in both group M and group C compared to group P at all the measured intervals, with the exception of the first hour following surgery.	Patients in groups M and C needed fewer additional pain medications and had lower rates of nausea and vomiting than those in group P. In summary, taking 6 mg of melatonin or 2 g of vitamin C orally before surgery reduced pain levels, the overall morphine use, the need for extra analgesia, and the frequency of nausea and vomiting compared to a placebo.	At peritoneal closure, the patients received a loading dose of 0.1 mg/kg of IV morphine (Galen Co) and 75 mg of intramuscular diclofenac sodium (Voltaren, Novartis) for postoperative pain control. After extubation, they were transferred to the PACU and monitored for at least 60 min. A PCA device delivering morphine at 0.3 mg/mL was then connected to a vein, enabling patient-controlled analgesia. The PCA was set to deliver 0.2 mg/kg bolus doses with a 10 min lockout and no background infusion. If a patient reported pain with a VAS score above 4 and requested additional relief beyond PCA, 75 mg of IM diclofenac sodium was given as supplemental analgesia.
Aboelela M., et al. (2023) [21]	Egypt	A randomized double-blind clinical trial	50	Laparoscopic sleeve gastrectomy	Vitamin C, 500 mg, as an oral capsule, or a placebo.	Every 8 h for 5 days perioperatively (4 days preoperative, and the operative day).	The VAS score was lower in group C compared to group N at 4, 8, and 12 h after surgery.	Vitamin C’s antioxidant ability, pain-relieving effects, and NMDA-receptor-blocking activity make it suitable for inclusion in multimodal pain strategies. Vitamin C serves as a co-analgesic, enhances postoperative pain control, and lowers the reliance on additional analgesics.	Morphine IV, 0.05–0.10 mg/kg, if the VAS score was >4.
Han G., et al. (2024) [22]	China	A prospective randomized double-blind trial	100	THA (endoprosthetics)	During surgery, the vitamin C group was administered 3 g of vitamin C diluted in 500 mL of normal saline via IV, while the control group received a 3 g placebo solution.	A total of 24 h after surgery.	Although the resting and movement-related pain scores were lower and hip mobility was improved at 24 h post-surgery in the vitamin C group, no statistically significant differences were observed between the groups at that time point. Moreover, the overall reductions in morphine use and the VAS scores did not exceed the minimal clinically important thresholds (10 mg for morphine; 1.5 at rest; and 1.8 with movement on the VAS).	The main objective was to assess whether giving intravenous vitamin C around the time of total hip replacement would lower postoperative morphine consumption. While the intake over the first 24 h after surgery was evaluated, no significant differences were found between the two groups during the overall hospital stay.	During the perioperative period, the surgeon administered periarticular local infiltration analgesia. If the patient was unable to endure the pain, 10 mg of morphine hydrochloride was administered subcutaneously as rescue analgesia.
Bala R., et al. (2024) [23]	England	A prospective randomized double-blind trial	60	Laparoscopic cholecystectomy	Group C (*n* = 30) was given 1 g of vitamin C (10 mL of 100 mg/mL Curevit) diluted with 40 mL of 0.9% NaCl to make 50 mL in total, while group N received 50 mL of saline alone.	If another dose was needed, it could be repeated after 8 h. As a third option, 50 mg of diclofenac was given intramuscularly, with a maximum dosing interval of once every 8 h.	On the NRS, a pain intensity > 4 was observed in group C immediately after surgery, as well as 4 and 24 h after the procedure.	A single intraoperative dose of vitamin C is a reasonable option in multimodal anesthesia for patients undergoing laparoscopic cholecystectomy, as it alleviates postoperative pain, reduces the need for rescue analgesia, and is safe and well tolerated.	Intraoperative analgesia was supplemented by fentanyl, 1 μg/kg/h, and port-site infiltration with a local anesthetic.
Sivro M., et al. (2024) [24]	Bosnia and Herzegovina	A prospective, single-blind, controlled, randomized clinical trial	60	Trochanteric fracture treated with intramedullary nailing	The vitamin C group received 2 g of ascorbic acid in 500 mL of 0.9% NaCl intravenously 30 min before the incision, followed by 1 g in 500 mL of saline daily for two days after surgery. The control group was given 500 mL of 0.9% NaCl without vitamin C, following the same schedule.	The median postoperative consumption of metamizole was significantly higher in the control group than in the vitamin C group (*p* = 0.003).	The visual analog scale (VAS) scores were assessed at 24 and 48 h after surgery. The median scores were significantly greater in the control group compared to the vitamin C group at both time points (*p* = 0.001 and *p* < 0.0005, respectively).	The findings indicated a marked decrease in reported pain and the reduced use of analgesics among patients given intravenous vitamin C, supporting its potential role as an adjunctive analgesic in elderly individuals with hip fractures.	Metamizole was given intravenously for pain relief, with a maximum allowable dose of 5 g per day.

**Table 4:** Summary of the studies on the analgesic effects of vitamin C.

	Vitamin C (p.o)	Vitamin C (IV)	Vitamin C + MgSO_4_
Vitamin C blood concentration	Low	High	High
Pain relief effect	Minimal	Moderate	Stronge (synergism)
Opioid consumption	No/minimal reduction	Moderate reduction	Reduction

## Data Availability

The authors declare that the data of this research are available from the corresponding author upon request.
